# The Question of Dairy Produce

**Published:** 1920-02-21

**Authors:** 


### THE QUESTION OF DAIRY PRODUCE.

The complexities and difficulties of the decontrol of food to which we recently alluded, and the results which fall upon the overburdened shoulders of the unhappy consumer are becoming still more obvious. British produce was recently freed from the straight waistcoat of control, and, as everybody could have foretold, the immediate effect was the doubling in price of home-made butter. In fact, it was gleefully announced some time ago that butter would probably rise to 5*s.* or 6*s.* a pound, as if foreknowledge of this pleasant fact would soften its application. There are various problems involved, and the points are not easily followed by inexpert students of domestic economy. The farmers and the wholesalers seem to be at loggerheads over prices, each accusing the other of feathering nests, but meanwhile the consumer receives mighty little consideration. The farmers of Leicestershire, for instance, are holding up milk supplies owing to the refusal of some wholesale sellers to pay the farmers their price, and the latter say they will convert their milk into butter, cream, and cheese. We have the off-repeated tale of the farmer's woes, as though he has not had the time of his life during recent years. He is always ready to detail his difficulties, and he now adds that of the provisions which enforce decent conditions of labour for his workmen. His past record has been so bad in this respect in many places —and still is so bad—that it is a topic he would be better advised to leave untouched. In remote parts of the country, the hours of labour and the pay of farm workers—male or female—are to say the least, unreasonable. It is not the provision of proper conditions that the public is anxious about, but the extent of profits. Has the farmer bought his motors—as so many of them have—on losses incurred by extra wages?

But the point of special concern in dairy produce is that it so deeply affects national health, that all producers and wholesalers ought to have some regard for that point of view. There are responsibilities as well as profits in farming. The State control of it has been aimed, with some success, at securing plentiful milk supplies, and every sort of generous safeguard has been provided for the producer in order to obtain it. Decontrol removes that supervision and leaves the producer to exploit the national health by putting his milk to the making and supply of butter at high prices to the rich and well-to-do. A shortage of milk, or an exorbitantly high price is a serious matter for the child-life of the nation, and if the producers cannot be persuaded of the evil consequences of their selfishness, the State ought not to remove its controlling hand.

If the State is prepared to let poor people suffer from lack of milk—the importance of which is being emphasised by every health association and in every official utterance on the subject—then it must reckon with a very nasty and dangerous temper, which may have results more far-reaching than in the economic field only. It is quite possible that decontrol inevitably leads to a rise in prices for a time, but conditions are not yet normal, and it is premature to give free-play to conscienceless tinkering with vital foodstuffs. The application of the profiteering regulations is feeble and ineffective, and it is the duty of the authorities either to continue control until a more favourable time, or rigidly and effectively to supervise profit-making in all directions. It is no use fixing the price of milk at such-and-such a price if it ends in a milk shortage because milk is applied to butter profiteering. Is there any reason, also, why butter should be denied to so many who have to continue with the less nutritious margarine? Whatever the facts are of our admittedly complicated situation, there is grave suspicion in the public mind that it is being fooled, and that it has to pay for the fooling. Nobody likes too much bureaucracy, which, in itself, is wasteful. What is wanted is proper care, ruthlessly exercised, in the interests of public wellbeing.

